# Protein Kinase Mitogen-activated Protein Kinase Kinase Kinase Kinase 4 (MAP4K4) Promotes Obesity-induced Hyperinsulinemia*

**DOI:** 10.1074/jbc.M116.718932

**Published:** 2016-05-20

**Authors:** Rachel J. Roth Flach, Laura V. Danai, Marina T. DiStefano, Mark Kelly, Lorena Garcia Menendez, Agata Jurczyk, Rohit B. Sharma, Dae Young Jung, Jong Hun Kim, Jason K. Kim, Rita Bortell, Laura C. Alonso, Michael P. Czech

**Affiliations:** From the ‡ Program in Molecular Medicine,; ¶ Department of Medicine, Division of Endocrinology, Metabolism and Diabetes, and; § Division of Cardiovascular Medicine, University of Massachusetts Medical School, Worcester, Massachusetts 01605

**Keywords:** diabetes, insulin, mitogen-activated protein kinase (MAPK), pancreas, pancreatic islet

## Abstract

Previous studies revealed a paradox whereby mitogen-activated protein kinase kinase kinase kinase 4 (Map4k4) acted as a negative regulator of insulin sensitivity in chronically obese mice, yet systemic deletion of Map4k4 did not improve glucose tolerance. Here, we report markedly reduced glucose-responsive plasma insulin and C-peptide levels in whole body Map4k4-depleted mice (M4K4 iKO) as well as an impaired first phase of insulin secretion from islets derived from M4K4 iKO mice *ex vivo*. After long-term high fat diet (HFD), M4K4 iKO mice pancreata also displayed reduced β cell mass, fewer proliferating β cells and reduced islet-specific gene mRNA expression compared with controls, although insulin content was normal. Interestingly, the reduced plasma insulin in M4K4 iKO mice exposed to chronic (16 weeks) HFD was not observed in response to acute HFD challenge or short term treatment with the insulin receptor antagonist S961. Furthermore, the improved insulin sensitivity in obese M4K4 iKO mice was abrogated by high exogenous insulin over the course of a euglycemic clamp study, indicating that hypoinsulinemia promotes insulin sensitivity in chronically obese M4K4 iKO mice. These results demonstrate that protein kinase Map4k4 drives obesity-induced hyperinsulinemia and insulin resistance in part by promoting insulin secretion from β cells in mice.

## Introduction

Highly controlled inter-organ networks operate to achieve proper blood glucose regulation, which is essential for mammalian survival (1). Pancreatic β cell function is a major contributor to control of glucose homeostasis by secreting the hormone insulin, which binds the insulin receptor to promote glucose uptake in peripheral tissues including muscle and adipose tissue while suppressing hepatic glucose production. In obesity, insulin often fails to adequately promote peripheral glucose uptake and suppress hepatic glucose production, resulting in hyperglycemia, which is associated with a state described as insulin resistance (2, 3). This insulin-resistant state promotes an increase in pancreatic β cell mass (4) and enhanced insulin secretion (5, 6) to normalize glucose levels, which results in hyperinsulinemia. Obese and insulin-resistant patients eventually display β cell dysfunction, resulting in an inability to control glycemia, and type 2 diabetes (T2D)^4^ ensues (5).

Interestingly, recent evidence suggests that although hyperinsulinemia is beneficial to restore euglycemia acutely, the actions of chronically high insulin levels can promote adipose tissue dysfunction and insulin resistance (7, 8). Treatments to improve peripheral insulin sensitivity, suppress hepatic glucose output, and potentiate pancreatic β cell insulin secretion are mainstays of T2D treatment. However, these treatments are often insufficient to fully control glucose homeostasis (9). T2D is an enormous burden on the health care system because of its association with a number of comorbidities including cardiovascular disease; thus further therapeutics are required.

Our laboratory identified the protein kinase mitogen-activated protein kinase kinase kinase kinase 4 (Map4k4) as a negative regulator of insulin-mediated glucose transport (10). We observed that inducible whole-body Map4k4 gene deletion in adult mice generated by crossing Map4k4-floxed mice to ubc-ERT2 cre mice improved insulin sensitivity but not glucose tolerance (11). By selectively deleting Map4k4 in different cell types *in vivo*, the increased insulin sensitivity in the whole body knock-out was attributed at least in part to Map4k4 within a myogenic factor 5 (myf 5)-positive lineage (11).

Here, we demonstrate that inducible, systemic loss of Map4k4 ameliorates long-term HFD-induced hyperinsulinemia. Mice lacking Map4k4 (M4K4 iKO) fed HFD for 16 weeks displayed reduced insulin secretion *in vivo* and a blunted β cell hypertrophic response. However, a short-term high fat diet (HFD; 4 weeks) or acute treatment of chow-fed control and M4K4 iKO mice with the peptide insulin receptor antagonist S961 induced similar hyperinsulinemia, suggesting that Map4k4 is required for chronic adaptation but not an acute response to systemic insulin resistance. The reduction in insulin levels in M4K4 iKO mice after chronic HFD was at least partially mediated by reduced insulin secretion from the β cell, as islets isolated from HFD-fed mice lacking Map4k4 demonstrated a reduced first phase of glucose-induced insulin secretion concomitant with reduced islet-specific gene expression. Thus, Map4k4 mediates hyperinsulinemia in chronic obesity by promoting islet hypertrophy and insulin secretion from pancreatic β cells.

## Results

### 

#### 

##### Mature Mice Inducibly Lacking Map4k4 Are Not More Insulin Sensitive in a Euglycemic Clamp

**FIGURE 1.**
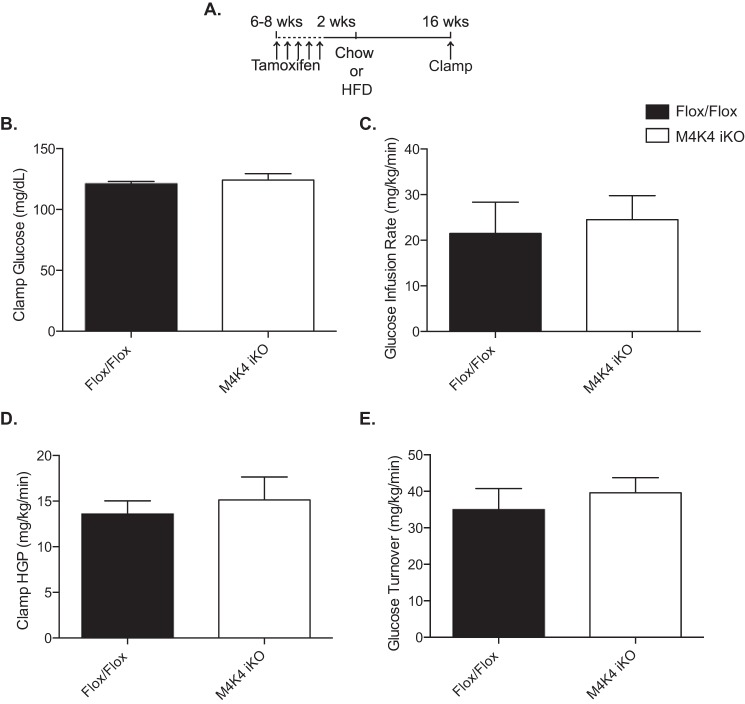
**Similar glycemic control in hyperinsulinemic Flox/Flox and M4K4 iKO mice.** Map4k4 Flox/Flox or Flox/Flox ubc-ERT2 cre animals were injected with tamoxifen for 5 days at 6–8 weeks of age. After 2 weeks, the mice were placed on chow or a HFD for 16 additional weeks. *A*, injection scheme. *B–D*, hyperinsulinemic euglycemic clamps were performed after a HFD for 16 weeks. *B*, clamp glucose. *C*, glucose infusion rate. *D*, hepatic glucose output. *E*, glucose turnover. (Data are mean ± S.E., *n* = 4, 6.)

Previous reports from our laboratory described whole body inducible Map4k4 knock-out mice, which were generated by crossing Map4k4 Flox/Flox mice (Flox/Flox) to a tamoxifen-inducible ubiquitin cre system (Ubc-ERT2 Cre) (11) (M4K4 iKO). Though they displayed similar body weight and fat mass, long-term high fat diet (HFD)-fed M4K4 iKO animals displayed enhanced peripheral insulin sensitivity in an ITT compared with Flox/Flox animals (11). To further characterize this insulin sensitivity phenotype, we subjected Flox/Flox and M4K4 iKO mice to a hyperinsulinemic-euglycemic clamp after 16 weeks of HFD (Fig. 1*A*), and no differences were observed between genotypes in clamped glucose levels (Fig. 1*B*), glucose infusion rate (Fig. 1*C*), hepatic glucose production (Fig. 1*D*), or glucose turnover (Fig. 1*E*). We had previously reported that insulin levels were dramatically reduced in HFD-fed M4K4 iKO mice compared with controls (11). We thus hypothesized that the matched, increased insulin levels during the hyperinsulinemic-euglycemic clamp may have masked the improved insulin sensitivity in M4K4 iKO mice, and M4K4 iKO mice may display enhanced insulin sensitivity in an ITT in part due to lower insulin levels during this test. We therefore sought to assess whether Map4k4 had a role within the islet to control insulin secretion.

##### Map4k4 Is Present in β Cells and Contributes to Chronic Obesity-induced Hyperinsulinemia

**FIGURE 2.**
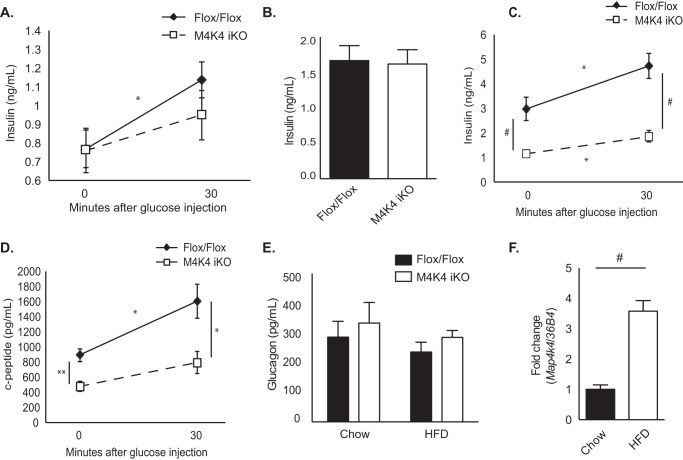
**Reduced insulin levels in HFD-fed M4K4 iKO compared with Flox/Flox mice.** Control Flox/Flox and M4K4 iKO mice were fed chow or HFD for 4 or 16 weeks post injections. *A–E*, Flox/Flox and M4K4 iKO mice were fasted overnight, and insulin or C-peptide levels were assessed basally and/or 30′ after 1 g/kg glucose injection. *A*, chow-fed insulin levels. *B*, 4 weeks HFD-fed insulin levels. *C*, 16 weeks HFD-fed insulin levels. *D*, 16 weeks HFD-fed C-peptide levels. *E*, plasma glucagon levels in overnight fasted chow- and 16 weeks HFD-fed animals. *F*, islets were isolated from Flox/Flox control mice, and qRT-PCR was performed for *Map4k4* and normalized to *36b4*. (*n* = 6–10, *, *p* < 0.05; **, *p* < 0.005; #, *p* < 0.0001). Data are mean ± S.E.

Fasting plasma insulin levels were unchanged in chow-fed M4K4 iKO mice compared with Flox/Flox controls (Fig. 2*A*). However, whereas insulin levels significantly increased in response to glucose injection in Flox/Flox animals (0.77 ± 0.10 *versus* 1.14 ± 0.10 ng/ml), there was no significant increase in M4K4 iKO mice (0.76 ± 0.12 *versus* 0.95 ± 0.13 ng/ml; Fig. 2*A*), suggesting that insulin secretion *in vivo* may be reduced or delayed in M4K4 iKO mice. Similar results were observed after Flox/Flox and M4K4 iKO animals were fed HFD for 4 weeks, as both Flox/Flox and M4K4 iKO mice demonstrated similar basal insulin levels (1.66 ± 0.21 ng/ml *versus* 1.62 ± 0.20 ng/ml; Fig. 2*B*), which were significantly increased from that observed in chow-fed animals (Fig. 2*A*). However, while 16 weeks of HFD promoted further hyperinsulinemia in Flox/Flox animals compared with chow-fed or 4 weeks HFD-fed counterparts (0.77 ± 0.10 ng/ml chow, 1.66 ± 0.21 ng/ml 4 weeks HFD, 2.96 ± 0.47 ng/ml 16 weeks HFD; Fig. 2, *A–C*), M4K4 iKO animals did not display HFD-induced hyperinsulinemia (0.76 ± 0.12 ng/ml chow, 1.15 ± 0.12 ng/ml 16 weeks HFD; Fig. 2, *A–C*). Furthermore, insulin levels significantly increased in Flox/Flox animals in response to an i.p. glucose injection, (2.96 ± 0.47 *versus* 4.72 ± 0.52 ng/ml; Fig. 2*C*), which was not evident in M4K4 iKO animals (1.15 ± 0.12 *versus* 1.86 ± 0.23 ng/ml; Fig. 2*C*). These data suggest that systemic loss of Map4k4 ameliorates chronic obesity-induced hyperinsulinemia *in vivo*.

To assess whether insulin levels were decreased in HFD-fed M4K4 iKO mice due to changes in insulin secretion, C-peptide levels were measured. C-peptide is secreted in an equimolar ratio to insulin and has a constant clearance rate; therefore, it is a useful parameter to assess insulin secretion by β cells as well as insulin clearance (14). Similar to insulin levels, 16 weeks HFD-fed M4K4 iKO mice displayed reduced C-peptide levels both basally and in response to a bolus glucose injection (891.9 ± 87.4 pg/ml *versus* 1602.4 ± 226.2 pg/ml, and 478.5 ± 67.95 pg/ml *versus* 792.88 ± 148.0 pg/ml, respectively; Fig. 2*D*). These data suggest that systemic Map4k4 is required for HFD-induced hyperinsulinemia.

Pancreatic islets are comprised of α and β cells, and while the β cell controls glucose-induced insulin secretion, the α cell controls fasting-induced glucagon secretion (15). However, no alterations in plasma glucagon levels were noted in chow-fed or HFD-fed Flox/Flox and M4K4 iKO mice after an overnight fast (Fig. 2*E*). These data suggest that Map4k4 is required for pancreatic β but not α cell function *in vivo* in obese mice.

To determine whether Map4k4 was indeed expressed in pancreatic islets *in vivo* and could potentiate hyperinsulinemia, islets were isolated from Flox/Flox animals fed chow or HFD, mRNA was extracted, and Map4k4 mRNA levels were assessed and normalized to *36b4*. A 3-fold increase in Map4k4 mRNA expression was noted in Flox/Flox animals fed HFD for 16 weeks compared with chow diet (Fig. 2*F*), suggesting that Map4k4 may contribute to HFD-induced hyperinsulinemia in an islet-specific manner.

##### M4K4 iKO Mice Can Effectively Secrete Insulin in Response to Acute Stress

**FIGURE 3.**
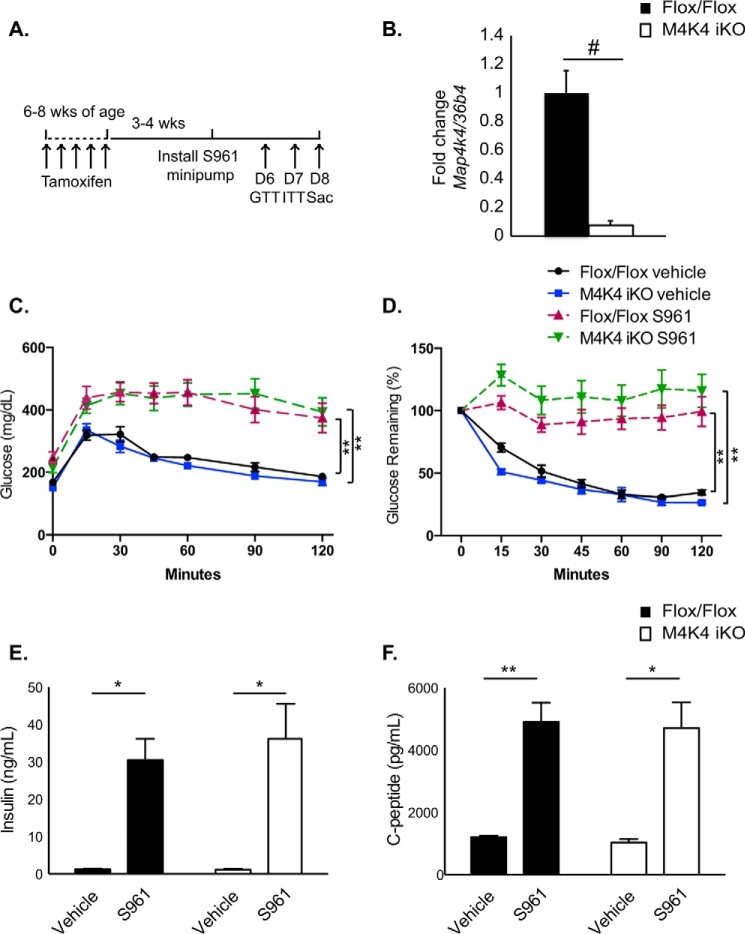
**Acute insulin resistance increases plasma insulin levels in Flox/Flox and M4K4 iKO mice.** 3–4 weeks after tamoxifen treatment, chow-fed Flox/Flox and M4K4 iKO mice were treated with 10 nmol of S961 or vehicle for 1 week by minipump. *A*, experimental timeline. *B*, islets were isolated on D8, and quantitative RT-PCR was performed for *Map4k4* and normalized to *36b4* (#, *p* < 0.0005; *n* = 5). *C*, mice were subjected to an i.p. GTT on day 6. *D*, mice were subjected to an i.p. ITT on day 7. *E*, plasma insulin levels were measured after a 4 h fast on day 8. *F*, plasma C-peptide levels were measured after a 4-h fast on day 8 (*, *p* < 0.05; **, *p* < 0.005; *n* = 5–6 vehicle, 11–12 S961). Data are mean ± S.E.

Insulin resistance promotes a compensatory increase in insulin levels via pancreatic β cell proliferation and enhanced insulin secretion to maintain homeostasis (16). Thus, to assess whether Map4k4 insulin secretion in M4K4 iKO animals could still respond to acute stress induced by insulin resistance, chow-fed Flox/Flox or M4K4 iKO animals were made systemically insulin resistant using the peptide insulin receptor antagonist S961, which indirectly as a consequence of insulin resistance potently induces β cell proliferation and insulin secretion (17, 18). Mice were implanted with a minipump subcutaneously 3–4 weeks after tamoxifen administration and treated with 10 nmol of S961 or vehicle for 1 week, and GTTs and ITTs were performed prior to sacrifice (Fig. 3*A*). Islets isolated from animals after S961 treatment demonstrated over 95% deletion of Map4k4 mRNA using this protocol (Fig. 3*B*).

We have previously reported that there were no differences in basal glucose tolerance or insulin sensitivity in chow-fed Flox/Flox and M4K4 iKO mice (11). Indeed, vehicle-treated M4K4 iKO animals displayed no differences in glucose tolerance or insulin sensitivity compared with Flox/Flox controls (Fig. 3, *C* and *D*). As has been previously reported (17), S961 treatment induced glucose intolerance and insulin resistance in both Flox/Flox and M4K4 iKO animals to a similar extent within a 7-day period (Fig. 3, *C* and *D*). After 1 week of treatment with S961, plasma insulin and C-peptide levels were assessed after a 4-h fast. In vehicle-treated mice, similar levels of plasma insulin (Fig. 3*E*) and C-peptide (Fig. 3*F*) levels were observed. However, while a long-term HFD caused hyperinsulinemia in Flox/Flox animals but not M4K4 iKO mice (Fig. 2), acute S961 treatment induced both plasma insulin as well as C-peptide levels to a similar extent in both genotypes (Fig. 3, *E* and *F*). Taken together, these data suggest that Map4k4 is not required to mediate hyperinsulinemia in response to acute stress but is required for maintaining insulin levels in long term HFD stress (Fig. 2).

##### Normal Islet Morphology and Gene Expression in M4K4 iKO Mice

**FIGURE 4.**
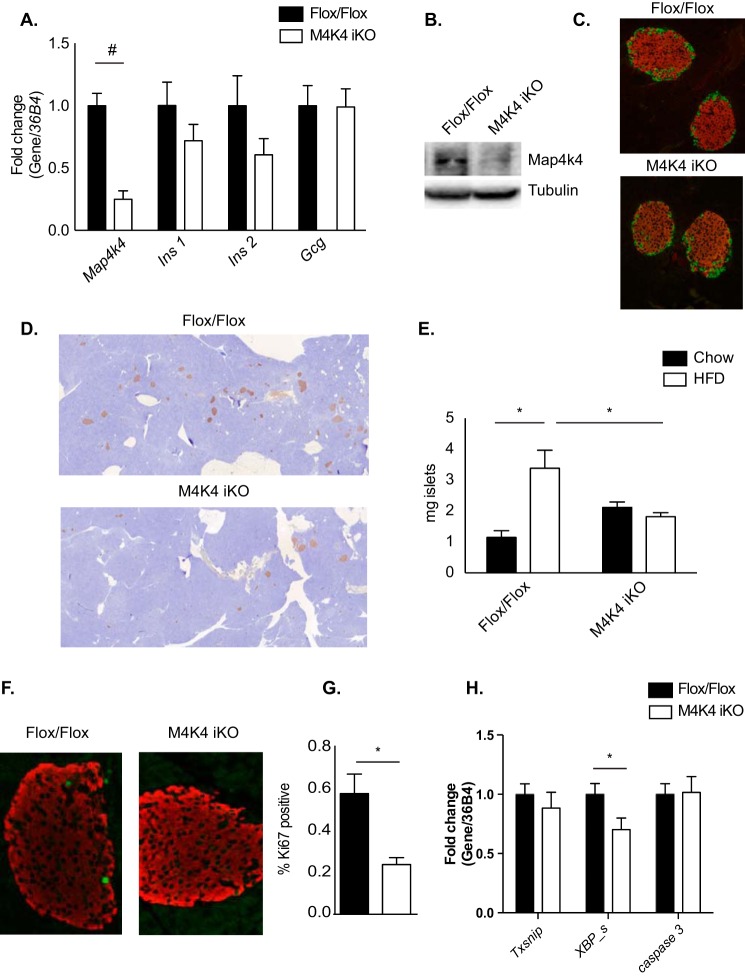
**Morphologically normal but reduced pancreatic islet mass in HFD-fed M4K4 iKO mice.** *A*, *C–H*, Flox/Flox and M4K4 iKO mice were fed HFD for 16 weeks following tamoxifen treatment. *A*, islets were isolated from HFD-fed mice, and quantitative RT-PCR was performed for the indicated genes and normalized to *36b4* (#, *p* < 0.0005; *n* = 10). *B*, reduced Map4k4 protein expression in whole pancreata derived from chow-fed Flox/Flox and M4K4 iKO mice. Data are representative of Flox/Flox and iKO mice. *C*, pancreata from HFD-fed mice were stained with insulin (*red*) and glucagon (*green*). Representative Flox/Flox and M4K4 iKO islets are shown at 20× magnification. *D* and *E*, pancreata were stained for insulin (*brown*) and hematoxylin (*blue*). *D*, representative images from HFD-fed mice. *E*, quantitation of islet mass as a percentage of total pancreas mass multiplied by pancreas weight. (ANOVA *, *p* < 0.05; *n* = 5–12). *F* and *G*, pancreata were stained with insulin (*red*) and Ki67 (*green*). *F*, representative islets. *G*, quantitation of the percentage of Ki67+/insulin+ cells (*, *p* < 0.05; *n* = 4–5). *H*, islets were isolated, and qRT-PCR was performed for the indicated genes and normalized to *36b4* (*, *p* < 0.05; *n* = 10). Data are mean ± S.E.

To better understand how Map4k4 ameliorated HFD-induced hyperinsulinemia, gene expression was next assessed in primary islets isolated from 16-week HFD-fed Flox/Flox or M4K4 iKO mice. Whereas a highly significant 70% reduction in Map4k4 gene expression was observed in islets and a similar reduction in Map4k4 protein expression was observed in immunoblots of whole pancreata that were isolated from M4K4 iKO mice (Fig. 4, *A* and *B*), no significant changes were observed in islet insulin (*Ins1*) or glucagon (*Gcgn*) mRNA levels, although a trend to a reduction in *Ins2* mRNA levels were observed (Fig. 4*A*). Similar islet morphology was also observed in 16-week HFD-fed Flox/Flox and M4K4 iKO pancreata as assessed by immunofluorescence for insulin (β cells) and glucagon (α cells) (Fig. 4*C*). These data suggest that loss of Map4k4 is not critical for general islet maintenance in obesity.

##### Reduced Islet Mass in Obese M4K4 iKO Mice

Loss of Map4k4 did not affect islet morphology in obese mice, but this does not exclude the possibility that Map4k4 may affect overall β cell mass or function. Thus, β cell mass was quantified by immunohistochemical staining for insulin as a percentage of pancreas mass in chow and 16 weeks HFD-fed Flox/Flox and M4K4 iKO animals. In chow animals, β cell mass was slightly increased (1.15 ± 0.22 *versus* 2.12 ± 0.17 mg; Fig. 4*E*). As expected, islet mass significantly increased after a 16-week HFD in Flox/Flox animals (1.15 ± 0.22 *versus* 3.39 ± 0.58 mg; Fig. 4*E*), whereas no such increase occurred in M4K4 iKO animals (2.12 ± 0.17 *versus* 1.82 ± 0.13 mg; Fig. 4*E*), which was a significant 76% reduction compared with Flox/Flox animals after 16-weeks of HFD (Fig. 4, *D* and *E*).

Increased islet mass may be mediated by β cell proliferation (16, 19, 20); thus, β cell proliferation rates were estimated by assessing the percentage of insulin-positive cells in HFD-fed animals that also expressed Ki67, a marker of proliferating cells, using immunofluorescence (Fig. 4*F*). Indeed, 16-week HFD-fed M4K4-iKO mice displayed a significant 58% reduction in the percentage of Ki67/insulin-positive cells compared with control mice (0.57% ± 0.09 *versus* 0.24% ± 0.03; Fig. 4*G*), consistent with the reduction that was observed in islet mass (Fig. 4, *D* and *E*). Furthermore, the reduction in islet mass was not likely due to enhanced cell death as the expression of apoptosis-associated genes *Txsnip*, *Casp 3*, or spliced XBP were not increased in islets that had been isolated from 16-week HFD-fed control or iKO mice (Fig. 4*H*). These results suggest that Map4k4 promotes HFD-induced β cell expansion by enhancing proliferation.

##### Reduced Insulin Secretion in Islets Derived from HFD-fed M4K4 iKO Mice

**FIGURE 5.**
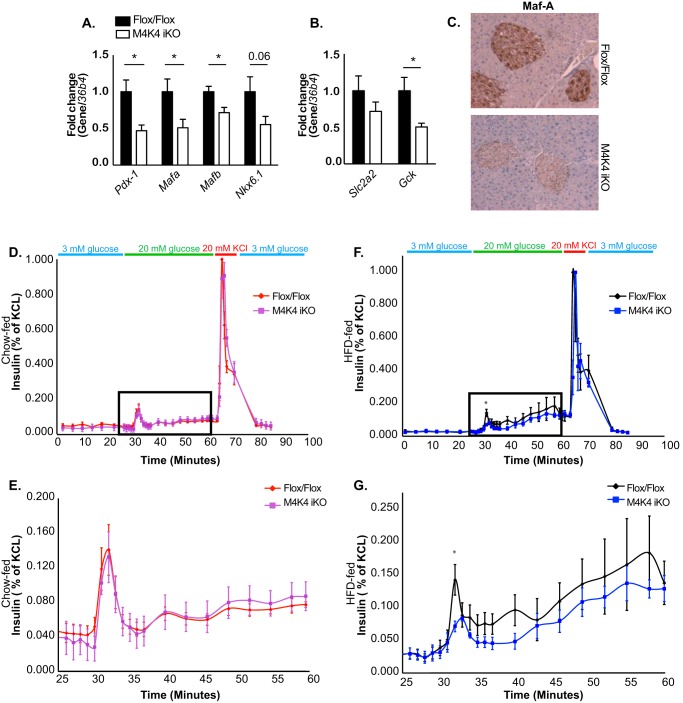
**Reduced isletspecific gene expression and insulin secretion in M4K4 iKO islets.** Flox/Flox and M4K4 iKO mice were fed HFD for 16 weeks following tamoxifen treatment. *A* and *B*, islets were isolated, and quantitative RT-PCR was performed for the indicated genes and normalized to *36b4* (*, *p* < 0.05; *n* = 10). *C*, pancreata were isolated and immunostained for Maf-A. Data are representative of 4 per genotype. *D–G*, islets were isolated, and glucose-induced insulin secretion was performed. Insulin levels are shown from isolated islets in response to 20 mm glucose and KCl as a percentage of total insulin released upon KCl treatment. *D* and *E*, chow fed animals, *E*, higher magnification of *square box* from *D*. *F* and *G*, HFD-fed animals. *G*, higher magnification of *square box* from *F* (*, *p* < 0.05; *n* = 4). Data are mean ± S.E.

In addition to islet mass, we assessed whether Map4k4 may also regulate islet function and insulin secretion. Thus, islets were isolated from HFD-fed Flox/Flox and M4K4 iKO animals, and qRT-PCR was performed for genes that are important for islet function. Consistent with a role for Map4k4 in β cell function, mRNA levels of islet-specific genes including Pancreatic and duodenal homeobox 1 (*Pdx-1*), v-maf musculoaponeurotic fibrosarcoma oncogene family, protein A (*Mafa*), and *Mafb* were significantly decreased, and levels of *Nkx6.1* were also reduced in islets derived from M4K4 iKO mice (Fig. 5*A*). The reduction in Maf A protein expression in M4K4 iKO islets was additionally confirmed by immunostaining pancreas sections from HFD-fed Flox/Flox or M4K4 iKO animals (Fig. 5*C*). Glucose sensing is also critical for insulin secretion, and several genes including glut-2 (*Slc2a2*) and glucokinase (*Gck*) are responsible for this response. Though no significant changes were observed in *Slc2a2* mRNA expression, *Gck* expression was significantly reduced in iKO mouse islets compared with those derived from Flox/Flox animals (Fig. 5*B*), suggesting that islets lacking Map4k4 expression may not properly sense the slow rise in glucose that occurs during HFD-induced obesity.

Glucose-induced insulin secretion was functionally assessed *ex vivo* in islets derived from chow or HFD-fed Flox/Flox and M4K4 iKO mice using a perifusion model in which medium was collected every 1–3 min to assess insulin secretion basally (3 mm glucose) as well as after the addition of 20 mm glucose or 20 mm KCl. As expected (21), high glucose concentrations enhanced insulin secretion in Flox/Flox islets in a biphasic manner, with a rapid peak (1^st^ phase) of insulin secretion occurring very shortly after introduction of 20 mm glucose at ∼minute 30, followed by a prolonged (∼minute 35 to 50) 2^nd^ phase of insulin secretion (Fig. 5, *D–G*). Interestingly, though islets derived from chow-fed M4K4 iKO mice displayed similar insulin secretion (Fig. 5, *D* and *E*), islets isolated from HFD-fed M4K4 iKO mice displayed a significant attenuation in this 1^st^ phase of insulin secretion (Fig. 5, *F* and *G*), which is one of the earliest detectable signs of β cell dysfunction in those destined to develop type 2 diabetes, which occurs prior to changes in plasma glucose levels (22–24). This reduction in the first phase of insulin secretion in HFD-fed M4K4 iKO mice is also a primary characteristic of β cell dysfunction. These data suggest that loss of Map4k4 reduces not only β cell mass (Fig. 4) but also impairs β cell function and glucose-induced insulin secretion in obese mice under long-term stress, possibly due to abnormal glucose sensing (Fig. 5).

## Discussion

Secretion of insulin by pancreatic β cells is critical to promote peripheral glucose uptake and maintain euglycemia when glucose levels rise (3). Insulin secretion can be regulated on a tissue level by increasing β cell mass and number through proliferation or on a cell autonomous level by altering β cell depolarization, or both (5). However, increased physiological demand for insulin, such as in insulin resistance, can lead to β cell dysfunction and type 2 diabetes (5). Therefore, it is necessary to understand the physiological signals that lead to demand-induced β cell proliferation and insulin secretion.

We had previously demonstrated that inducible, systemic loss of Map4k4 in adult mice improved insulin sensitivity (11). Here, we expand upon these observations with the unexpected finding that this improved sensitivity is at least in part due to reduced insulin levels in M4K4 iKO mice. Thus, under hyperinsulinemic euglycemic clamp conditions, no difference in glucose infusion rates between M4K4 iKO mice and controls in the context of hyperinsulinemia were observed (Fig. 1). These data indicate that hyperinsulinemia over the course of these clamp studies reverses the increased insulin sensitivity induced by chronically low insulin levels in M4K4 iKO mice.

We sought to explain the reduced insulin levels observed after HFD in M4K4 iKO mice. Short-term (4 weeks) HFD as well as acute hyperinsulinemia mediated by systemic insulin resistance in response to S961 treatment did not reveal a marked difference in insulin levels between genotypes (Figs. 2 and 3), suggesting that long-term adaptive mechanisms rather than a primary defect in secretion may be more important in establishing the reduced insulin phenotype observed in HFD-fed M4K4 iKO mice. In support of this hypothesis, reduced β cell mass and proliferation were observed in M4K4 iKO mice after long-term HFD (Fig. 4). Furthermore, expression of genes that determine β cell specification and insulin secretion were reduced in islets that had been isolated from obese mice lacking Map4k4 (Fig. 5), suggesting that islet-specific Map4k4 is important for mediating long-term adaptation to metabolic stress.

Mice lacking systemic Map4k4 displayed reduced β cell hypertrophy in obesity compared with Flox/Flox counterparts, at least in part due to reduced HFD-induced β cell proliferation (Fig. 4). Recent studies suggest that β cell proliferation is required for β cell expansion in obesity and in times of increased insulin demand because most β cell expansion is mediated by self-replication (16, 19, 20). In obesity, several physiological signals including neuronal inputs (25), microvascular dysfunction (26), and paracrine hormonal regulation (27) are also important for β cell function on a systemic level. Because the mice described herein utilize ubiquitin cre to delete Map4k4 systemically, a specific cell type that is mediating these effects on insulin levels is difficult to delineate. Important future studies will include defining a cell-autonomous role for Map4k4 in the pancreatic β cell using mouse insulin promoter (Mip-1) cre animals (28).

It is unclear why Map4K4 iKO mice displayed similar insulin levels after acute challenge to the pancreas such as in 4 weeks of HFD or after S961 treatment. It is possible that factors related to the high lipid content of HFD may mediate these effects on the β cell, as β cells are susceptible to lipotoxicity (29). Another possibility is that the acute insulin resistance stimulus mediated by S961 treatment was too short to observe a more adaptive effect in M4k4 iKO mice. Perhaps a sub-maximal dose of S961 treatment over a longer time period would have a different effect on insulin secretion in this context.

Previous studies have implicated a cell autonomous role for Map4k4 in glucose-induced insulin secretion *in vitro* using a rat β cell line. Bouzraki *et al.* (30) suggested that deletion of Map4k4 ameliorated the TNF-α-induced decrease in glucose-induced insulin secretion *in vitro*, and Zhao *et al.* (31) suggested that Map4k4 is a target of miRNA-30d, which induces insulin gene expression in β cells. Both of these studies report that Map4k4 expression is inhibitory to insulin gene expression and insulin secretion, yet our *in vivo* studies reveal that loss of Map4k4 in islets of HFD-fed mice actually reduced insulin secretion from β cells *ex vivo*. Furthermore, while Bouzakri *et al.* demonstrated that loss of Map4k4 in cultured β cells induced *Mafa* and *Pdx-1* gene expression (30), Map4k4 iKO mice demonstrated reduced *Mafa* and *Pdx-1* gene expression in primary islets (Fig. 5*A*). Interestingly, we also observed enhanced *Pdx-1* and *Mafa* gene expression upon Map4k4 silencing in the Min6 β cell line, similar to Bouzakri *et al.* (not shown). These results suggest that there are fundamental differences in the roles of Map4k4 in pancreatic islets and β cell culture lines, which will be explored in future studies.

The mechanism by which Map4k4 regulates plasma insulin levels remains unclear. Other studies have implicated the related ste-20-like kinase MST-1 as a positive regulator of β cell apoptosis in diabetes, and loss of MST-1 promoted normoglycemia in diabetic animal models (32). In our studies, β cell apoptosis was assessed both by gene expression analysis (Fig. 4) and TUNEL staining (data not shown), and little to no apoptosis was observed in either control or Map4k4 iKO mice using these methods. β cell failure in diabetes is also attributed to de-differentiation of β cells into other endocrine cells (33). However, glucagon and insulin staining in M4K4 iKO islets after long-term HFD revealed normal and uniform staining (Fig. 4), suggesting that Map4k4 loss is not promoting islet de-differentiation. Thus, future studies will be required to determine the molecular targets of Map4k4 that mediate its function in β cells.

In summary, we have demonstrated that Map4k4 is required for chronic HFD-induced hyperinsulinemia in mice. Map4k4 promoted islet expansion in obesity, and islets derived from Map4k4 iKO mice demonstrated reduced insulin secretion *ex vivo* (Fig. 5). These data support a complex role for Map4k4 in physiology to regulate not only glucose uptake and insulin sensitivity, but also the secretion of insulin itself and further suggest that Map4k4 is a critical protector against β cell failure in chronic overnutrition.

## Experimental Procedures

### 

#### 

##### Animal Studies

All of the studies performed were approved by the Institutional Animal Care and Use Committee (IACUC) of the University of Massachusetts Medical School. Map4k4 Flox/Flox-UBC-cre ERT2 animals and tamoxifen dosing have been previously described (11). Animals were maintained in a 12 h light/dark cycle. Mice were fed chow or HFD (Research diets 12942i) for 4 or 16 weeks starting 2 weeks post tamoxifen injections. Mice were euthanized by CO_2_ inhalation followed by bilateral pneumothorax.

##### Glucose and Insulin Tolerance Tests

HFD-fed mice were fasted for 16 h for a glucose tolerance test (GTT) or for 4 h for an insulin tolerance test (ITT). S961-treated mice were fasted for 4 h before both tests. Fasted mice were i.p.-injected with glucose (1 g/kg) or insulin (1 IU/kg). *In vivo* insulin secretion studies were assessed from blood drawn from the tail vein 30′ after glucose injection. Blood glucose levels were determined using a Breeze-2 glucose meter (Bayer).

##### S961 Experiments

3–4 weeks post tamoxifen treatment, chow-fed mice were anesthetized with isoflurane, and Alzet 2001 minipumps (Alzet) containing 10 nmol of S961 peptide (Phoenix Pharmaceuticals) or equivalent vehicle (5% DMSO in PBS) were implanted subcutaneously for 1 week. A GTT and ITT were performed after a 4-h fast on days 6 and 7, respectively, and mice were sacrificed on day 8 after a 4-hour fast.

##### Hyperinsulinemic Euglycemic Clamp Studies

The clamp study was performed by the National Mouse Metabolic Phenotyping Center at UMass. 16-week HFD-fed mice were subjected to an overnight fast, and a 2-h hyperinsulinemic-euglycemic clamp was conducted in awake mice with a primed and continuous infusion of human insulin (150 mU/kg body weight priming followed by 4 mU·kg^-1^ min^-1^; Humulin, Eli Lilly). During the clamp, 20% glucose was infused at variable rates to maintain euglycemia (12).

##### Quantitative RT-PCR

RNA was isolated from primary islets, and quantitative RT-PCR was performed as previously described (11). Primer sequences are as follows: *36b4* (5′-TCCAGGCTTTGGGCATCA-3′, 3′-CTTTATCAGCTGCACATCACTCAGA-5′); *Map4k4* (5′-CATCTCCAGGGAAATCCTCAGG-3′, 3′-TTCTGTAGTCGTAAGTGGCGTCTG-5′); *Ins1* (5′-CCATCAGCAAGCAGGTCATTG-3′, 3′-TGTGTAGAAGAAGCCACGCTCC-5′); *Ins2* (5′-CAGAAACCATCAGCAAGCAGG-3′, 3′-TTGACAAAAGCCTGGGTGGG-5′); *Gcgn* (5′-GAGGAACCGGAACAACATTGC-3′, 3′-GCAATGAATTCCTTTGCTGCC-5′); *Slc2a2* (5′-TGCTGCTGGATAAATTCGCC-3′, 3′-TCAGCAACCATGAACCAAGG-3′); *Mafa* (5′-AGGAGGAGGTCATCCGACTG-3′, 3′-CTTCTCGCTCTCCAGAATGTG-5′); *Mafb* (5′-AGGACCTGTACTGGATGGC-3′, 3′-CACTACGGAAGCCGTCGAAG-5′); *Nkx6.1* (5′-CTTCTGGCCCGGAGTGATG-3′, 3′-GGGTCTGGTGTGTTTTCTCTTC-5′); *Pdx-1* (5′-GAGGTGCTTACACAGCGGAA-3′, 3′-GGGGCCGGGAGATGTATTT-5′), *gck,* (5′-TGAGCCGGATGCAGAAGGA-3′, 3′ AGGCCAGTGTAAAGATGTTGC-5′), *txsnip* (5′-TCCTCAAGATGGGTGGCAAT-3′, 3′-GGCGTTGTCTTTCCAAGGTT-5′), *xbp_s* (5′-CTGAGTCCGAATCAGGTGCAG-3′, 3′-CCAGAACATCTTCCCATGGAC-5′), *Casp3* (5′-CAGCCAACCTCAGAGAGACA-3′, 3′-TCACACACACAAAGCTGCTC-5′).

##### Plasma Biochemical Analysis

Mice were fasted overnight or for 4 h, and plasma was collected via tail vein or cardiac puncture. Plasma insulin, C-peptide, and glucagon levels were assessed using insulin (Millipore), C-peptide (ALPCO), or glucagon (R&D systems) ELISAs, respectively, according to the manufacturer's instructions. Glucagon levels were measured immediately upon blood collection. Insulin levels from perifusion analysis and isolated islets were also measured by ELISA as above.

##### Histology

Mouse pancreata were isolated and fixed in 10% formalin for 4 h followed by paraffin embedding and sectioning. Slides were stained with hematoxylin, insulin (abcam ab7842 or ab6995), glucagon (abcam ab10988), Ki67 (abcam ab16667 or ab6526), or Maf-A (ab17976). The ratio of β-cell area to total pancreas area was calculated from one or the average of 2 histological images (one superficial, one deep) using BioPix software by selecting the insulin-positive (brown) area as a percentage of total pancreas area (blue) and multiplying by the wet weight of the pancreas to obtain β cell mass in mg.

##### Western Blotting Analysis

Whole pancreata were harvested and snap frozen in liquid nitrogen followed by lysis and sonication (150 mm Tris, 2% SDS, 1% Triton, 5 mm EDTA, 2× HALT protease, and phosphatase inhibitors (Thermo Pierce)). SDS-PAGE gels were run and transferred to nitrocellulose membranes. Membranes were immunoblotted with anti-Map4k4 (Bethyl) or -tubulin (Sigma) antibodies.

##### Islet Isolation and Insulin Secretion

Islets were isolated from non-fasted animals by collagenase digestion and Ficoll-histopaque density gradient separation as described (13). Glucose-stimulated insulin secretion was measured using a BioRep perifusion system for which 25 similarly shaped and sized islets were hand-selected using a microscope. Islets were incubated in 3 mm glucose for 25 min followed by stimulation with 20 mm glucose for 40 min and finally 20 mm KCl for 5 min prior to returning to 3 mm glucose. Medium was collected every 1–3 min to assess insulin secretion. Secreted insulin in response to glucose was normalized to KCl-mediated insulin secretion.

##### Statistical Analysis

A two-tailed Student's *t* test was used to compare two groups in Microsoft Excel. Where indicated, experiments comparing multiple groups were analyzed by ANOVA in Graph Pad Prism 6.0. *p* < 0.05 was considered to be statistically significant. Variance was estimated using the standard error of the mean.

## Author Contributions

R. J. R. F., L. V. D., L. C. A., M. P. C., J. K. K., and R. B. designed the studies, R. J. R. F., L. V. D., M. K., M. T. D., A. J., D. Y. J., J. H. K., and R. S. performed research, and R. J. R. F., L. V. D., and M. P. C. wrote the paper.

## Footnotes

4 The abbreviations used are:

T2D: type 2 diabetes
Map4k4: mitogen-activated protein kinase kinase kinase kinase 4
M4K4 iKO: Map4k4-depleted mice
HFD: high fat diet.
